# Maximum Urine Flow Rate of Less than 15ml/Sec Increasing Risk of Urine Retention and Prostate Surgery among Patients with Alpha-1 Blockers: A 10-Year Follow Up Study

**DOI:** 10.1371/journal.pone.0160689

**Published:** 2016-08-11

**Authors:** Hsin-Ho Liu, Tsung-Hsun Tsai, Shang-Sen Lee, Yu-Hung Kuo, Tengfu Hsieh

**Affiliations:** 1 Department of Urology, Taichung Tzu Chi General Hospital, Buddhist Tzu Chi Medical Foundation, Taichung, Taiwan; 2 School of Medicine, Tzu Chi University, Hualian, Taiwan; 3 Department of Research, Taichung Tzu Chi General Hospital, Buddhist Tzu Chi Medical Foundation, Taichung, Taiwan; Taipei Medical University, TAIWAN

## Abstract

**Background:**

The aim of this study was to determine the subsequent risk of acute urine retention and prostate surgery in patients receiving alpha-1 blockers treatment and having a maximum urinary flow rate of less than 15ml/sec.

**Methods:**

We identified patients who were diagnosed with benign prostate hyperplasia (BPH) and had a maximum uroflow rate of less than 15ml/sec between 1 January, 2002 to 31 December, 2011 from Taiwan’s National Health Insurance Research Database into study group (n = 303). The control cohort included four BPH/LUTS patients without 5ARI used for each study group, randomly selected from the same dataset (n = 1,212). Each patient was monitored to identify those who subsequently developed prostate surgery and acute urine retention.

**Results:**

Prostate surgery and acute urine retention are detected in 5.9% of control group and 8.3% of study group during 10-year follow up. Compared with the control group, there was increase in the risk of prostate surgery and acute urine retention in the study group (HR = 1.83, 95% CI: 1.16 to 2.91) after adjusting for age, comorbidities, geographic region and socioeconomic status.

**Conclusions:**

Maximum urine flow rate of less than 15ml/sec is a risk factor of urinary retention and subsequent prostate surgery in BPH patients receiving alpha-1 blocker therapy. This result can provide a reference for clinicians.

## Introduction

Taiwan implemented its national health insurance scheme 20 years ago [1, 2]. Today, the National Health Insurance (NHI) Administration covers 99.7% of Taiwan’s population, and governs treatment modalities for Taiwan’s physicians. Under this insurance scheme, medical institutions provide treatment and then apply to the NHI for reimbursement. Therefore, NHI regulations have considerable influence, and medical practices which diverge from NHI regulations are ineligible for reimbursement, and may also incur penalties.

For example, take the treatment of benign prostate hyperplasia with lower urinary tract syndrome (BHP with LUTS, NHI regulations provide that patients with a maximum urine flow rate of less than 15ml/sec for whom alpha-1 blockers are ineffective can receive 5- alpha reductase inhibitors (5ARI) treatment [3, 4]. Taiwan urologists have been observing this provision for 20 years. The uroflow study is the most useful, objective and noninvasive method of assessing bladder outflow obstruction [5–7]. However, no large-scale and long-term follow up studies have been conducted to determine whether the 15ml/sec requirement is reasonable or not, especially in patients whom alpha-1 blockers are ineffective.

In this study, we use data from the NHI database to determine whether a maximum urinary flow of 15ml/sec is a relatively high degree of acute urine retention (AUR) for BPH with LUTS patients, or whether it indicates an acceptable risk for prostate surgery. In recent study demonstrated that the data from NHI database can be used as a general comorbidity measure to describe the health status of populations based on data derived from population-based automated health care databases[8]. The findings may provide clinicians with a reference for weighing treatment options, and can also provide a reference for the determination of public health treatment policies.

## Material and Method

### Ethics Statements

The Institutional Review Board of Taichung Tzu Chi General Hospital in Taiwan approved the study protocol (REC103-43). Because the identification numbers and personal information of the individuals in this study were not included in the secondary files, the review board waived the need for written consent.

### Data Source

This study used NHIRD records from 1 January, 2002 to 31 December, 2011. The NHIRD is maintained by Taiwan’s National Health Research Institutes, and is made available to researchers (http://nhird.nhri.org.tw/date_01_en.html).

### Study Design

In our study, the International Classification of Diseases, 9th Revision, Clinical Modification (ICD-9-CM) diagnosis codes and ICD-9-CM treatment codes were evaluated. The defined daily dose (DDD) is a unit for measuring a prescribed amount of a drug; it is the assumed average daily maintenance dose of a drug consumed for its main indication in adults [9]. The cumulative DDD (cDDD), which indicates the duration of exposure, was estimated as the sum of dispensed DDD of 5-alpha-reductase inhibitor.

All patients with newly-diagnosed BPH (ICD-9-CM code 600.xx) and followed-up between 1 January, 2003 to 31 December, 2011 were included. In Taiwan, a physician makes a diagnosis of BPH on an outpatient basis must include digital rectal examination and clinical symptoms reported by patients [10]. In order to increase the validity of the BPH diagnoses, only those patients who had received three or more BPH diagnoses was included in this study. The study excluded patients with newly-diagnosed acute urine retention, who had received a transurethral resection of prostate (TURP) or had urine retention before the index date, who had a follow-up duration of less than 6 months.

According to NHI regulation, 5ARI only can be used in patients with maximum urine flow rate of less than 15ml/sec, moderate to severe signs and symptoms of bladder outlet obstruction (BOO), excluding the possibility of prostate cancer and failure of alpha one blocker treatment. Patients who received 5ARI inhibitor therapy for less than 28 cDDD and had a uroflowmetry study before 5ARI therapy were identified as the patients with maximum urine flow rate of less than 15ml/sec. These patients were identified as study group. The date of initiation of 5ARI therapy was used as the patient’s index date.

The control cohort included four BPH patients without 5ARI used for each BPH patient with 5ARI used, randomly selected from the same dataset to reduce selection bias by bundling many confounding covariates that may be present in an observational study with this number of variables.

Independent variables were gender, co-morbid disorders, geographical area of residence, urbanization level, and socioeconomic status. S1 Fig displays a flowchart diagram explaining the numbers of individuals at each stage of the study.

### Research Outcomes

The main outcome of the study was the occurrence of prostate surgery (ICD-9-CM Code: 60.21 and 60.29), which was determined by linking records with inpatient care data in the NHIRD or acute urine retention, which was determined by linking records with inpatient care data or out-patient service claims of urine retention diagnosis (ICD-9-CM Code: 788.2) and urethral catheterization (47013C, 47014C). All patients in this study had a wash-out period of six months after index data.

### Other Variables

The Charlson Comorbidity Index Score (CCIS) is a widely accepted measure for risk adjustment in administrative claims data sets [11–14]. The CCIS were calculated for each patient by assigning 1 point each for myocardial infarct, congestive heart failure, peripheral vascular disease, dementia, cerebrovascular disease, chronic lung disease, connective tissue disease, ulcer, chronic liver disease and diabetes by assigning 2 points each for hemiplegia, moderate or severe kidney disease, diabetes with end organ damage, tumor, leukemia and lymphoma, 3 points for moderate or severe liver disease and 6 points each for malignant tumor, metastasis and acquired immune deficiency syndrome.

The study subjects were classified into three groups: (1) low socioeconomic status (<US$528 per month); (2) moderate socioeconomic status (US$528–833 per month); and (3) high socioeconomic status (≥US$833 per month) [15]. Low income was set at the equivalent of US$528 as this has been the government-stipulated minimum wage for full-time employees in Taiwan since 2006 [15]. Geographic region of residence was recorded as northern, central, southern, and eastern Taiwan. The regions where the individuals resided in Taiwan were classified into 7 levels of urbanization based on 5 indices: population density, percentage of residents with college level or higher education, percentage of residents > 65 years of age, percentage of residents who work in agriculture, and the number of physicians per 100,000 residents [16]. The urbanization level of residences were categorized as urban (urbanization level: 1), sub-urban (urbanization level 2–3), or rural (urbanization level 4–7).

### Statistical Analysis

SPSS version 15 (SPSS Inc., Chicago, IL, USA) was used for all data analyses. Pearson’s chi-square test was used for categorical variables such as gender, SES, geographic region of residence, and co-morbidities. Continuous variables were analyzed using one-way analysis of variance (ANOVA). The cumulative risk of prostate surgery or urine retention for study subjects was estimated using Kaplan-Meier survival curves.

A Cox proportional hazards regression model adjusted for patient characteristics (age, gender, co-morbidities, SES, and geographic region) was used to analyze the association of 5ARI use with subsequent TURP or urine retention during the 10-year follow-up period. Hazard ratios (HRs) and 95% confidence intervals (CIs) were calculated using the multivariate Cox proportional hazards regression model to evaluate the association between 5ARI used and the incidence of TURP or urine retention. Statistical significance was set at a two-sided p<0.05.

## Results

We had 22687 patients with newly diagnosis of BPH in our study. For explaining the numbers of individuals at each stage of the study, we draw a flowchart and display as supplementary figure (S1 Fig). A total of 1515 patients were included in our study, of whom 303 patients had maximum urine flow rate of less than 15ml/sec (study group) and 1212 did not (control group). The demographic characteristics and selected morbidities for the two cohorts are shown in Table 1. The average age in each group was 67.9 years. Except CCIS and diabetes, the two groups were consistent in terms of demographic characteristics, alpha-1 adrenergic blocker usage and selected morbidities. The study group had higher CCIS and more diabetes than control group. We have analyzed the frequent medical/urology visit and expense of each urological/medical visit into S1 Table. These are no different in urological/medical visit and cost of all medical visits, and little increase of cost of urological visit (863 NTD/year) in control group.

**Table 1:** Demographic Characteristics and co-morbidity (*n* = 1515).

Variables	Control		Qmax<15ml/sec	
	N	%	N	%
**Patient no.**	1212		303	
**Mean age, years(±SD)**	67.9±9.7		67.9±9.7	
**CCIS score(±SD)**	0.9±1.2		1.1±1.4*	
0–1	909	75.0	222	73.3
2–3	269	22.2	56	18.5
> 3	34	2.8	25	8.3
**Comorbidity**				
Diabetes	195	16.1	68	22.4**
Stroke	107	8.8	33	10.9
Multiple sclerosis	0	0	0	0
Parkinsonism	16	1.3	7	2.3
UTI	112	9.2	39	12.9
**α1- adrenergic blockers used**				
Only Non-selectiveα1- adrenergic blockers used	283	23.3	70	22.4
Only Selectiveα1- adrenergic blockers	258	21.3	72	23.0
Both	671	55.4	161	51.4
**Socioeconomic status**				
Low	414	34.2	108	35.6
Moderate	364	30.0	100	33.0
High	434	35.8	95	31.4
**Urbanization**				
Urban	426	35.1	90	29.7
Suburban	498	41.1	136	44.9
Rural	288	23.8	77	25.4
**Geographic region**				
Northern/Central	834	68.8	211	69.6
Southern/Eastern	378	31.2	92	30.4

# Non-selectiveα1- adrenergic blockers: terazosin、doxazosin、afluzosin、phenoxybenzamine; selective α1- adrenergic blockers: tamsulosin

* p<0.05

** P<0.01

*** p<0.001

Chi-square test; One-way ANOVA

**Fig 1:**
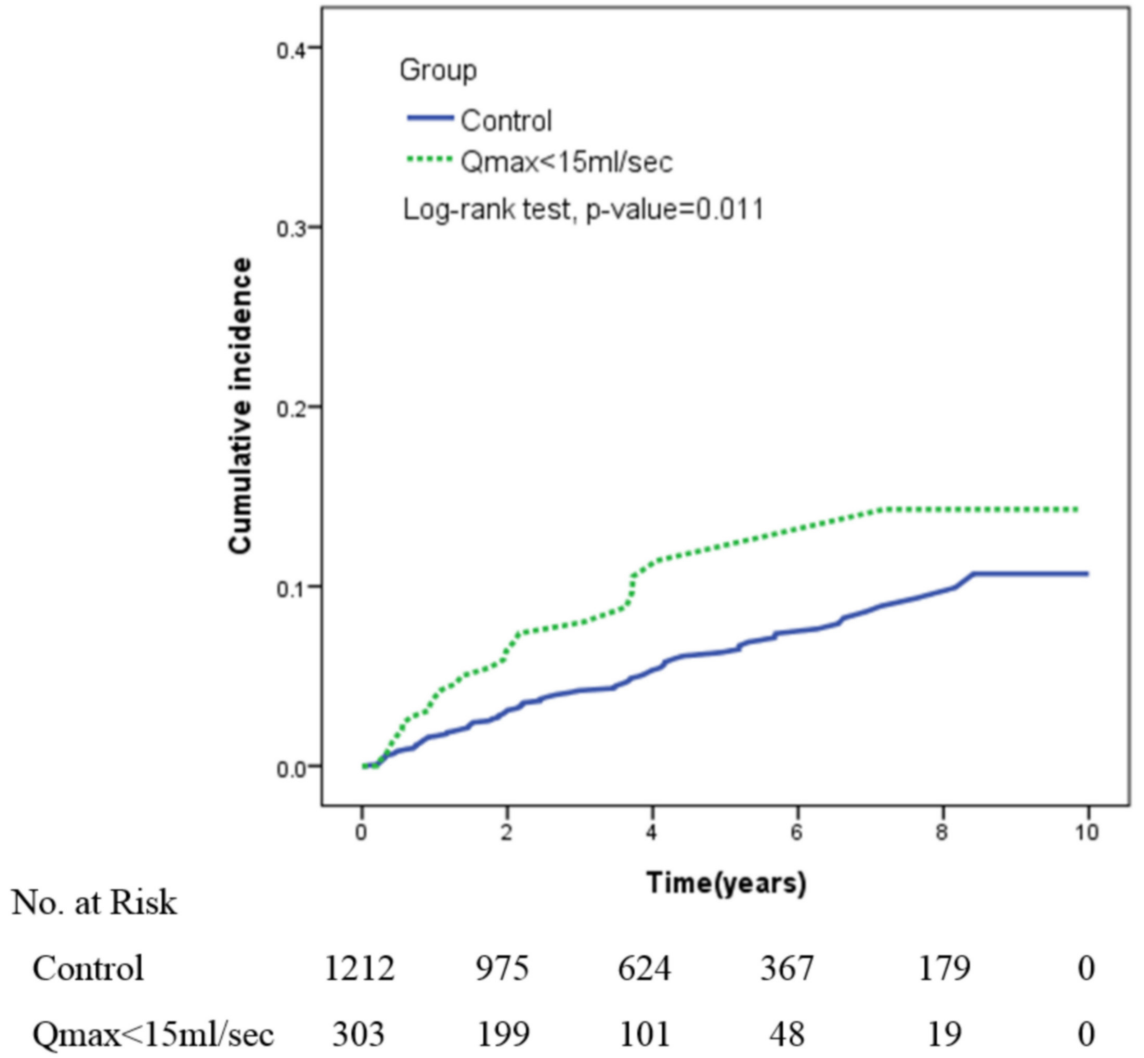
The cumulative incidence of prostate surgery and urine retention estimated by Kaplan-Meier method in two groups.

At the end of the follow-up period, 44 (2.9%) patients had prostate surgery, including 34 (2.8%) in the control group and 10(3.3%) in the study group. Moreover, 53 (3.5%) patients had urine retention, including 38 (3.1%) in the control group, 15(6.4%) in the study group (Table 2). As shown in the Kaplan-Meier curve in Fig 1, the 10-year risk of developing AUR and prostate surgery was consistent for two groups (log-rank test p = 0.011).

**Table 2:** The event number of prostate surgery and urine retention in patients.

Group	Prostate surgery		Urine retention		Total	
	N	%	N	%	N	%
**Control**	34	2.8	38	3.1	72	5.9
**Qmax<15ml/sec**	10	3.3	15	5.0	25	8.3

When adjusted for age and comorbidities, multivariate Cox proportional hazard regression analysis revealed the study group had a higher risk of developing urine retention and prostate surgery (Table 3). The adjusted HR was 1.83 (95% CI: 1.16 to 2.91) for the study group. Moreover, we found that age (HR = 1.04, 95% CI: 1.01 to 1.06) and patients in southern and eastern of Taiwan (HR = 1.57, 95% CI: 1.01 to 2.43) are another significant factor for incidence of prostate surgery and urine retention.

**Table 3:** Cox model measured hazard ratio and 95% confidence intervals.

Characteristics	Crude		Adjusted	
	HR	95% CI	HR	95% CI
**Group**				
Control	1	ref	1	Ref
Qmax<15ml/sec	1.79	1.14–2.83*	1.83	1.16–2.91*
**Age**	1.03	1.01–1.05**	1.04	1.01–1.06**
**CCIS score**	1.15	0.99–1.33	1.11	0.94–1.32
**Comorbidity**				
Diabetes				
No	1	ref	1	ref
Yes	1.39	0.86–2.25	1.18	0.70–2.00
Stroke				
No	1	ref	1	ref
Yes	0.90	0.44–1.86	0.67	0.31–1.44
UTI				
No	1	ref	1	ref
Yes	0.95	0.48–1.89	0.91	0.46–1.82
**Socioeconomic status**				
Low	1	ref	1	ref
Moderate	0.80	0.49–1.32	0.83	0.48–1.44
High	0.92	0.58–1.47	1.31	0.77–2.23
**Urbanization**				
Urban	1	ref	1	ref
Suburban	0.94	0.58–1.51	0.83	0.51–1.36
Rural	1.38	0.84–2.28	1.20	0.68–2.12
**Geographic region**				
Northern/Central	1	ref	1	ref
Southern/Eastern	1.58	1.06–2.36*	1.57	1.01–2.43*

Abbreviation: HR, hazard ratio; CI, confidence interval

Adjusted HR: adjusted for 5ARI dosage, age, CCIS, Socioeconomic status, comorbidity of Diabetes, Stroke and UTI in Cox proportional hazards regression

* p<0.05

** P<0.01

*** p<0.001

## Discussion

Our study finds that, even among patients receiving alpha-1 blocker therapy, a maximum urinary flow of less than 15ml/sec has an 83% higher risk of acute urinary retention and subsequent prostate surgery. This suggests that treatment of BPH with LUTS patients should seek to prevent the maximum urinary flow fall below 15ml/sec.

In Taiwan’s NHI system, 5ARI treatment is only available to patients with a maximum urinary flow rate below 15ml/sec and who do not respond to alpha-1 blockers. In this study, we use this rule to define 5ARI therapy patients as having a maximum urinary flow rate of 15ml/sec. To eliminate the influence of 5ARI drugs, we select patients who had only received short-term 5ARI treatment (<28 cDDD), followed by a six month washout period. We then observed patient urine retention and subsequent prescription of prostate surgery.

A key goal of treatment for BPH with LUTS is to prevent urinary retention and the need for prostate surgery [17]. Urinary retention can have a significant impact on patient quality of life, and the effectiveness of drug therapy following prostate surgery to treat urinary tract syndrome is less than ideal [18]. These two results are often used as indicators for the clinical evaluation of BPH with LUTS.

Theoretically, BPH with LUTS has 2 physiological components: a static component related to increased prostate size and a dynamic component related to increased prostate smooth muscle tone [19]. Apha-1 blockers relax prostate smooth muscle and decrease urethral resistance, ultimately leading to relief of LUTS. 5ARI therapy is well documented to reduce prostate size and LUTS symptoms [20, 21]. Apha-1 blockers and 5ARI therapy are the majority of medical treatment of BPH with LUTS. Many studies reported the combination with apha-1 blockers and 5ARI therapy to be effectiveness against BPH with LUTS ^8,20^.

Under normal circumstances, physicians prescribe different treatments depending on the specific condition of the patient. However, in Taiwan, treatment is guided by NHI regulations, and 5ARI therapy is only available to patients with a maximum urinary flow rate of 15ml/sec and who have responded poorly to alpha-1 blocker [3, 4]. Because 99.7% of Taiwan’s population is covered by the NHI system, the NHI database provides a highly uniform study population over the course of 10 years.

Despite the 10-year study period, there are several issues which are not addressed:

First, our research results suggest that a maximum urinary flow of 15ml/sec carries a high risk of urinary retention and subsequent prostate surgery. However, from the NHI database we are unable to determine which maximum urinary flow rate is the critical point. We know that graphic representations of urinary flow are of considerable help in determining urinary function[22–24]. However, these graphic representations cannot be obtained from the NHI database.

Also, while 5ARI therapy is only available to patients with a maximum urinary flow of 15ml/sec, BPH with LUTS is a progressive illness and, once the condition deteriorates to the point at which surgery is required, the results are generally poor. Is 15ml/sec already over the critical point? At that flow rate, is 5ARI treatment effective? Future work will seek to address these questions.

Third, this study found that for patients with a maximum urinary flow rate of 15ml/sec, patient age and residence in southern and eastern Taiwan are all risk factors for urinary retention and prostate surgery. Patient age is already a widely recognized risk factor [18]. However, patients residing in southern and eastern Taiwan are at greater risk for urinary retention and prostate surgery than those living in the north and west. We speculate this may have to do with the relative distribution of medical resources, as the southern and eastern regions are relatively poorly served, thus residents in these areas are less likely to receive early diagnosis and drug treatment, thus leading to increased rates of urinary retention and subsequent prostate surgery. This pattern has been seen in studies of other diseases. Of course, a large-scale public health study would be required to confirm this hypothesis.

Finally, our study population consisted entirely of patients who had undergone alpha-1 blocker treatment. Therefore, the higher risk of urinary retention and subsequent prostate surgery for BPH with LUTS patients with a maximum urinary flow rate of 15ml/sec cannot be generalized to patients who had not undergone alpha-1 blocker treatment. However, for the BPH with LUTS group, alpha-1 blocker is a first-line treatment modality, thus our results still have good clinical value.

## Conclusion

This study concludes that the BPH with LUTS patients receiving alpha-1 blocker therapy and having a maximum urinary flow rate of 15ml/sec are at higher risk of urinary retention and subsequent prostate surgery. This result can provide a reference for clinicians.

## Supporting Information

S1 Fig Flowchart diagram: explain the numbers of individuals at each satge of the study. (TIF)

S1 Table Use and costs of healthcare services within study by patients with Qmax less than 15ml/sec and comparison subjects (n = 1515). (DOC)
