# Supplementary figures and images for: Maximum Urine Flow Rate of Less than 15ml/Sec Increasing Risk of Urine Retention and Prostate Surgery among Patients with Alpha-1 Blockers: A 10-Year Follow Up Study
Source: PLoS One. 2016 Aug 11;11(8):e0160689. doi: 10.1371/journal.pone.0160689 (PMC4981394; doi:10.1371/journal.pone.0160689)

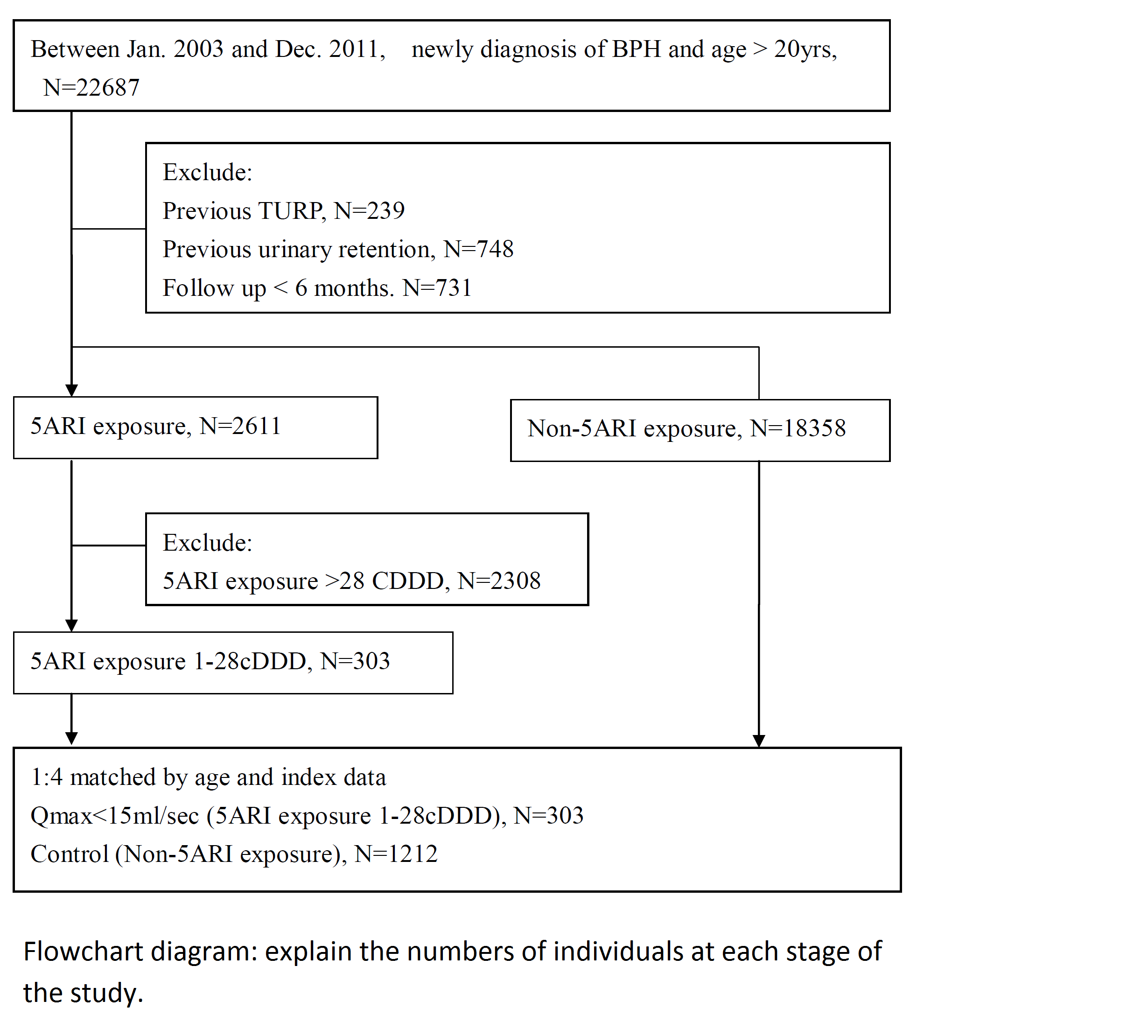

Supplement: S1 Fig — (TIF) [file pone.0160689.s001.tif]
