# Supplementary material for: Maximum Urine Flow Rate of Less than 15ml/Sec Increasing Risk of Urine Retention and Prostate Surgery among Patients with Alpha-1 Blockers: A 10-Year Follow Up Study
Source: PLoS One. 2016 Aug 11;11(8):e0160689. doi: 10.1371/journal.pone.0160689 (PMC4981394; doi:10.1371/journal.pone.0160689)
Supplement: S1 Table — (DOC) [file pone.0160689.s002.doc]

**S1 table** Use and costs of healthcare services within study by patients with Qmax less than 15ml/sec and comparison subjects (n = 1515).

| Variables | Control | | Qmax<15ml/sec, | | p-value* |
| --- | --- | --- | --- | --- | --- |
|  | **median** | **Q1,Q3** | **median** | **Q1,Q3** |  |
| Outpatient service |  |  |  |  |  |
| All outpatient visits/year, n | 33.1 | 21,49.3 | 32.8 | 21.5,48.5 | 0.959 |
| Cost of all outpatient visits /year, NTD | 31843.4 | 19099.1,53304 | 31650.6 | 19551.9,51450.4 | 0.182 |
| Urologic outpatient visits/year, n | 3.4 | 1.3,8.1 | 2.6 | 0.9,8.2 | 0.959 |
| Cost of urologic outpatient visits/year, NTD | 4136.1 | 1534.1,9291.2 | 3273.7 | 1084.6,9688.2 | 0.025 |
| Inpatient service |  |  |  |  |  |
| All admission/year, n | 0.2 | 0,0.6 | 0.2 | 0,0.8 | 0.269 |
| Cost of all admission/year, NTD | 18420 | 0,109350.5 | 16800 | 0,95155 | 0.004 |
| Admission to urologic ward/year, n | 0 | 0,0 | 0 | 0,0.2 | 0.217 |
| Cost of admissionto urologic ward/year, NTD | 0 | 0,0 | 0 | 0,6448.3 | 0.004 |

*Wilcoxon Signed-Rank Median Test

NTD, New Taiwan Dollar (1 USD = 32.5NTD)
